# Calpain-3-mediated regulation of the Na^+^-Ca^2+^ exchanger isoform 3

**DOI:** 10.1007/s00424-015-1747-8

**Published:** 2015-10-27

**Authors:** Lauriane Y. M. Michel, Joost G. J. Hoenderop, René J. M. Bindels

**Affiliations:** From the Department of Physiology, Centre for System Biology and Bioenergetics, Radboud University Medical Center, PO Box 9101, 6500 HB Nijmegen, The Netherlands

**Keywords:** Sodium-calcium exchange, Alternative splicing, Calpain-3, Muscular dystrophy, Muscle fatigue, Exercise

## Abstract

Ca^2+^ disturbances are observed when Ca^2+^-dependent cysteine proteases malfunction, causing muscle weakness and wasting. For example, loss of calpain-3 (CAPN3) activity leads to limb-girdle muscular dystrophy 2A (LGMD2A). In neuronal excitotoxicity, the cleavage of the Na^+^-Ca^2+^ exchanger isoform 3 (NCX3) has been associated with an increase in activity and elevation of the Ca^2+^ content in the endoplasmic reticulum (ER). Since NCX3 is expressed in skeletal muscle, we evaluated the cleavage of different NCX3 splice variants by CAPN1 and CAPN3. Using Fura-2-based cellular Ca^2+^ imaging, we showed for the first time that CAPN3 increases NCX3 activity and that only NCX3-AC, the variant predominantly expressed in skeletal muscle, is sensitive to calpain. The silencing of the endogenous CAPN1 and the expression of the inactive form of CAPN3 (C129S CAPN3) confirmed the specificity for CAPN1 and CAPN3. Functional studies revealed that cellular Ca^2+^ uptake through the reverse mode of NCX3 was significantly increased independently of the mode of activation of the exchanger by either a rise in intracellular Ca^2+^ ([Ca^2+^]_i_) or Na^+^ ([Na^+^]_i_). Subsequently, the sensitivity to CAPN1 and CAPN3 could be abrogated by removal of the six residues coded in exon C of NCX3-AC. Additionally, mutation of the Leu-600 and Leu-601 suggested the presence of a cleavage site at Leu-602. The increased Ca^2+^ uptake of NCX3 might participate in the Ca^2+^ refilling of the sarcoplasmic reticulum (SR) after the excitation-contraction uncoupling following exercise and therefore be implicated in the impaired reticular Ca^2+^ storage observed in LGMD2A.

## Introduction

The calpain proteases are Ca^2+^-dependent cysteine proteases expressed throughout the entire body. A compromised function of one of the calpain family members can lead to diverse pathologies such as embryonic lethality, gastropathy, muscular dystrophy, or platelet dysfunction [13]. In the human genome, 15 members of the calpain family have been found (CAPN1 to CAPN15) [47]. The distinctive characteristic of calpain lies in its activation by a rise in intracellular Ca^2+^ levels ([Ca^2+^]_i_). This phenomenon triggers the autolysis of the protease. The cytosolic concentration of Ca^2+^ required for this activation differs between isoforms. The targets of its proteolysis are difficult to predict as the calpain family recognizes a tertiary structure and not the primary sequence unlike many conventional proteases [29].

Contrary to CAPN1 and CAPN2 that are ubiquitously expressed, the third isoform CAPN3 is restricted to the skeletal muscle. Interestingly, nanomolar levels of Ca^2+^ are sufficient to activate CAPN3 while CAPN1 and CAPN2 require micromolar and millimolar concentrations, respectively [5]. In physiological conditions, CAPN3 is activated when local Ca^2+^ levels are higher than 200 nM over a long period. Such requirements are met following eccentric contractions [32, 33]. However, in tetanic and concentric contractions, CAPN3 remains in its inactive state, which makes CAPN3 activity specific to certain types of contractions.

CAPN3 dysfunction causes the limb-girdle muscular dystrophy (LGMD) 2A also called calpainopathy, which is the most common type of LGMD, with 30 % of the patients [39]. The LGMD2A is an autosomal recessive form of muscular dystrophy characterized by muscle weakness of the proximal limb-girdle muscles eventually leading to a loss of ambulation. It derives from a single mutation of CAPN3 causing a defect in CAPN3 function. As of now, 97 distinct mutations have been identified as a cause of LGMD2A [40].

Among many peculiarities, CAPN3 is not freely present in the cytoplasm like CAPN1 and CAPN2 but tightly bound to the N2 region of the myofiber [22] and to the triad [23] formed by a central t-tubule and two terminal cisternae of the sarcoplasmic reticulum (SR). In this region where many Ca^2+^ transporters are present, a tight control of the Ca^2+^ fluxes is maintained during excitation-contraction coupling by connecting the SR to the sarcolemma [1]. The absence of CAPN3 or its impaired function is linked with Ca^2+^ disturbances within the myofibers including a decrease in Ca^2+^ release from the SR [23]. Such impairments could well contribute to the clinical phenotype of the LGMD2A. Therefore, the investigation of the role of CAPN3 on the Ca^2+^ fluxes occurring at the triad is of great interest.

Recently, the Na^+^-Ca^2+^ exchanger family (NCX) has been shown to be a substrate of CAPN1 and CAPN2 [4]. The third member of the family, NCX3, was revealed to be the only isoform specifically cleaved during excitotoxic conditions [4]. NCX cleavage modifies its activity and the resulting Ca^2+^ influx [37]. Interestingly, NCX3 is also expressed in the skeletal muscles and has been found at the triad [41] where it extrudes Ca^2+^ across the sarcolemma prior to relaxation. Recently, NCX3 was hypothesized to be involved in the regulation of Ca^2+^ beyond Ca^2+^ extrusion, and several studies have suggested a role for NCX3 in Ca^2+^ re-entry into the SR [11, 30] during prolonged exercise.

The expression of *NCX3* can give rise to different variants [38] due to an alternative splicing. Two variants have been described in mice NCX3-AC and NCX3-B. Previous work from our group showed that these variants have different capacities of exchange in physiological situations [30]. It is however unclear whether the calpain family cleaves both variants and how the cleavage affects their functional properties.

In the present study, we investigated for the first time the sensitivity of the different NCX3 variants to calpain using Fura-2-based Ca^2+^ imaging in presence of the calpain inhibitor calpastatin. The results were confirmed by the use of a siRNA targeting specifically CAPN1. By expressing both CAPN3 and its inactive form (C129S CAPN3), a novel regulation of NCX3 variants by the skeletal muscle CAPN3 was found. These properties were further compared with the expression pattern of the two variants among the different myofiber types. Finally, the combination of data concerning the reverse mode triggered by [Ca^2+^]_i_ and by [Na^+^]_i_, together with the use of site-directed mutagenesis, enabled a full characterization of the transport capacities of the exchanger upon regulation by calpain and provided a new insight into the molecular determinants responsible for this sensitivity.

## Experimental procedures

### Animal model

Four C57BL/6 mice were sacrificed, and subsequently, the skeletal muscles from the left and right hind limbs were dissected and directly frozen in liquid nitrogen in order to obtain the gastrocnemius (Gast), the tibialis anterior (TA), the extensor digitorum longus (EDL), and the soleus. The heart sample used as a negative control was removed from four C57BL/6 mice.

### Cell lines and transfection

The human embryonic kidney cells HEK293T were cultured in a Dulbecco’s modified Eagle’s medium (Bio Whittaker-Europe, Verviers, Belgium) containing 10 % (*v*/*v*) fetal calf serum and 2 mM l-glutamine at 37 °C with 5 % (*v*/*v*) CO_2_. Forty-eight hours prior to experiments, cells were seeded on a six-well plate and transiently transfected. For double transfections, cells received 1 μg of the each constructs for a total of 2 μg/well, using polyethylenimine cationic polymer PEI (Polysciences Inc., Warrington, PA) in accordance with the manufacturer’s instructions.

### Cloning and mutagenesis

Coding sequence of the human calpastatin (hCAST) was obtained from Dharmacon Research Inc. (Lafayette, CO). Human calpain-3 (hCAPN3) (kindly provided by Dr. van der Maarel, Leiden, The Netherlands) was subcloned into a pCINeo IRES-mCherry backbone. The subcloning of the mice NCX3-AC and NCX3-B into pCINeo IRES-GFP vectors with a HA-Tag fused at the N-terminus of the protein has been previously described in detail [30]. Calpain cleavage sites were predicted using the *group base prediction system-calpain cleavage detector* (GPS-CCD) 1.0 prediction tool [27]. The variant-specific cleavage site was mutated using site-directed mutagenesis, performed on NCX3-AC leading to the following mutation: LL-600601-WW NCX3-AC targeting the cleavage sites. Additionally, the exon C consisting of the ALLLSP region could be removed to NCX3-AC (NCX3-A) and the C129S mutation inserted in hCAPN3 using a Quick-change site-directed mutagenesis kit (Stratagene, La Jolla, CA).

### Expression profile and quantitative real-time polymerase chain reaction analysis

Tissue RNA was extracted using TRIzol total RNA Isolation Reagent (Life Technologies BRL, Breda, The Netherlands). After DNAse treatment (Promega, Madison, WI), 1.5 μg of RNA was reverse-transcribed by Moloney-Murine Leukemia Virus-Reverse Transcriptase (Invitrogen, Carlsbad, CA) as previously described [15]. Using a CFX96 real-time PCR detection system (Bio-Rad, Hercules, CA), the relative quantification of NCX3 and the calpain isoforms were measured in the cDNA from tissues and normalized according to the Livak method on GAPDH expression. Absolute quantification of the variants NCX3-AC and NCX3-B was calculated thanks to NCX3 standard curves generated by using a diluted pCINeo-IRES-eGFP-mNCX3-AC and pCINeo-IRES-eGFP-mNCX3-B vectors. Calculations were performed as previously described [30, 51] in order to obtain the absolute copy number for each variant. Sequences were amplified using the validated primers described in the table below (Table 1).

**Table 1:** Primers sets for relative and absolute quantification by RT-PCR

Name of primers	Direction	Primer sequence 5′→3′
Total mNCX3	Forward	GAGCTGGAGTTCAAGAATG
Total mNCX3	Reverse	CTTCCTGTCTGTCACTTC
mNCX3-AC	Forward	GAAACAGTCAAAACAATTCACATC
mNCX3-AC	Reverse	GTCACTTCTGGAGATAACAGGAG
mNCX3-B	Forward	GTCACTTCTGGAGATAACAGGAG
mNCX3-B	Reverse	CTGTCACTTCTGATATTCC
mCAPN1	Forward	ACCACATTTTACGAGGGCAC
mCAPN1	Reverse	GGATCTTGAACTGGGGGTTT
mCAPN2	Forward	ACATGCGTACTCTGTCACCG
mCAPN2	Reverse	GCTGGGGCAATTGTCATTCC
mCAPN3	Forward	ACAACAATCAGCTGGTTTTCACC
mCAPN3	Reverse	CAAAAAACTCTGTCACCCCTCC
mGAPDH	Forward	TAACATCAAATGGGGTGAGG
mGAPDH	Reverse	GGTTCACACCCATCACAAAC

Each set of primers amplified a fragment of 70 to 150 bp and has been tested and validated beforehand

### Silencing of calpain-1

Downregulation of CAPN1 expression was performed using siRNA targeting human calpain-1 and consisting of three to five target-specific 19–25 nt siRNAs (Santacruz, Dallas, TX) and a control siRNA (Dharmacon Research Inc., Lafayette, CO). Twenty-four hours after plating, HEK293T cells were transfected with an optimized concentration of 30 nM of siRNA and 1 μg/well of the NCX3 construct, NCX3-AC or NCX3-B, and 1 μg/well of mock vector using Lipofectamine. Functional experiments were performed 72 h after transfection, time required for an optimal downregulation with such siRNA.

### Immunoblot analysis

HEK293T cells were lysed at 4 °C in a solution of 150 mM NaCl, 5 mM EGTA, 50 mM Tris, pH adjusted to 7.5 with NaOH, Triton X-100 0.5 % (*v*/*v*), and protease inhibitors (1 mM PMSF, 5 μg/mL leupeptin, 1 μg/mL aprotinin, 1 μg/mL pepstatin). After centrifugation at 12,000×*g* for 10 min, 5× Laemmli buffer containing 100 nM dithiothreitol was added to the supernatant. Lysates were subjected to SDS–PAGE 8 % (*w*/*v*) and electroblotted onto polyvinylidene difluoride (PVDF) membranes. Blots were incubated with 5 % (*w*/*v*) non-fat dried milk in TBS-T (137 mM NaCl, 0.2 % (*v*/*v*), Tween-20, and 20 mM Tris/HCl, pH 7.6). Immunoblots were incubated overnight at 4 °C with either a mouse anti-beta-actin antibody (1:1,000) (Sigma, MO, USA) or a goat anti-calpain-1 (1:150) (Santacruz, Dallas, TX) diluted in 1 % (*w*/*v*) milk in TBS-T. PVDF membranes were incubated 1 h at room temperature with a sheep horseradish peroxidase-conjugated anti-goat (1:2,000) or anti-mouse (1:10,000) antibody (Sigma, MO, USA) in TBS-T. Afterwards, blots were visualized using the enhanced chemiluminescence system (ECL, Thermo Fisher).

### Fura-2 measurements

NCX3 activity was measured by recording intracellular Ca^2+^ level. HEK293T cells were seeded on coverslips coated with fibronectin (Roche, Mannheim, Germany) prior to transfection. Forty-eight hours after transfection, the cells were loaded for 20 min at 37 °C with the ratiometric probe Fura-2-AM 3 μM and 0.01 % (*v*/*v*) pluronic acid F-127 (Invitrogen, Carlsbad, CA) in Krebs solution (5.5 mM KCl, 147 mM NaCl, 1.2 mM MgCl_2_, 1.5 mM CaCl_2_, 10 mM glucose, and 10 mM HEPES/NaOH, pH 7.4). After a 10-min wash, coverslips were mounted onto a stage of an inverted microscope (Zeiss Axiovert 200M, Carl Zeiss, Jena, Germany). Changes in medium and addition of compounds were facilitated using a perfusion system. [Ca^2+^]_i_ was monitored by exciting Fura-2 with monochromatic light of wavelength 340 and 380 nm (Polychrome IV, TILL Photonics, Gräfelfing, Germany). Fluorescence emission was directed by a 415DCLP dichroic mirror (Omega Optical, Inc., Brattleboro, VT) through a 510WB40 emission filter (Omega Optical, Inc.) onto a CoolSNAP HQ monochrome charge-coupled device (CCD) camera (Roper Scientific, Vianen, The Netherlands). The integration time of the CCD camera was set at 200 ms with a sampling interval of 3 s. All hardware was controlled with Metafluor software (version 6.0, Universal Imaging Corp., Downingtown, PA). For each wavelength, the mean fluorescence intensity was monitored in an intracellular region and, for purpose of background correction, in an extracellular region of identical size. After background correction, the fluorescence emission ratio (340 nm/380 nm) was calculated to determine the Fura-2 ratios. Ten to 19 individual GFP-positive cells were selected and monitored simultaneously from each coverslip. After double transfection of CAPN3 and NCX3, only cells positive for both GFP and mCherry were selected. The reverse mode of NCX activity was evaluated using the Ca^2+^ uptake that follows the removal of extracellular Na^+^ using a NMDG solution (5.5 mM KCl, 147 mM *N*-methyl glucamine, 1.2 mM MgCl_2_, 1.5 mM CaCl_2_, 10 mM glucose, and 10 mM HEPES/HCl, pH 7.4) [43]. All buffers were kept at 37 °C and the variability in osmolality was lower than 5 mOsm. The activation of the reverse mode of the exchanger was achieved by either raising the [Ca^2+^]_i_ using the sarcoplasmic reticulum Ca^2+^-ATPase inhibitor thapsigargin (1 μM) simultaneously with the Na^+^ removal or raising the [Na^+^]_i_ with a 60-min incubation in presence of the inhibitor of the Na^+^-K^+^ ATPase ouabain (1 μM) [43] prior to the experiments with a perfusion during recordings.

### Statistical analysis

All results are based on at least three different sessions of experiments. The Fura-2 ratio is an average of ≥50 individual cells. Values are expressed as means ± SEM. Statistical significance (*P* < 0.05) was determined using one-way ANOVA with the Bonferroni’s procedure.

## Results

### Expression of calpains and NCX3 in skeletal muscle

**Fig. 1:**
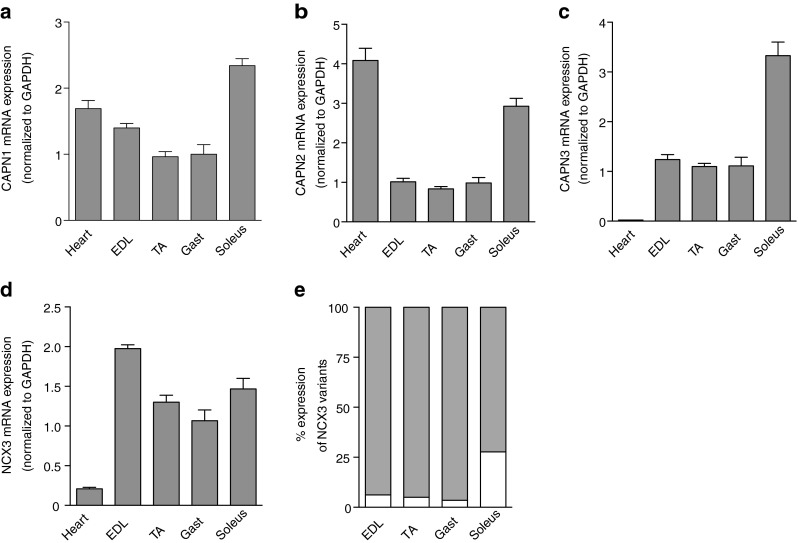
NCX3 and calpain expression in mice skeletal muscle. **a** CAPN1, **b** CAPN2, **c** CAPN3, and **d** NCX3 expression at mRNA levels in several types of skeletal muscle. Values are normalized on the housekeeping gene GAPDH. **e** The percentage of each NCX3 variants represented as the absolute copy number of either NCX3-B (*white*) or NCX3-AC (*gray*) compared to the sum of both quantifications. For clarity, SEM of value <3 % in each case is not depicted

Using RT-PCR, mRNA levels of the calpain family members were measured in cardiac tissues and skeletal muscles of different fiber types: slow-twitch fibers predominant in the soleus, fast-twitch fibers dominant in the extensor digitorum longus (EDL), or mixed fibers such as those found in the gastrocnemius and the tibialis anterior (TA). The measurements revealed that calpains were highly expressed in soleus, showing 2.3-, 2.9-, and 3.3-fold increases for CAPN1, CAPN2, and CAPN3, respectively, compared to the other skeletal muscles (Fig. 1a–c). Expression of CAPN1 in the heart was somewhat lower with a 1.3 fold-increase expression compared to the gastrocnemius, while CAPN2 was increased by a factor of 4.1. Finally, CAPN3 expression in cardiac tissue was more than 55 times lower than in skeletal muscle. A similar expression pattern was observed for NCX3, which confirmed the specific enrichment of both CAPN3 and NCX3 in the skeletal muscle (Fig. 1d). NCX3 expression was highest in the EDL, followed by the soleus, the TA, and the gastrocnemius. Using an absolute quantification method, we were able to obtain the percentage of the different variants of NCX3, NCX3-AC containing both exons A and C, and NCX-B containing only the exon B (Fig. 1e). These data revealed the predominance of NCX3-AC in all fiber types. However, in the soleus, NCX3-AC was 72 % of the total NCX3, while for the other fiber type, this percentage was higher than 94 %.

### Effect of CAPN on NCX3 activity

The effect of calpain activation on NCX3 reverse activity was investigated using the ratiometric Fura-2 probe. The reverse mode of exchange, where Ca^2+^ enters the cell, was triggered by the switch to a Na^+^-free medium together with a rise in either [Na^+^]_i_ or [Ca^2+^]_i_. In order to elevate the Ca^2+^ levels, the cells were perfused with a Na^+^-free medium containing thapsigargin. Thapsigargin inhibits the SERCA ATPase and therefore prevents the re-uptake of Ca^2+^ in the endoplasmic reticulum (ER). As the ER constitutively leaks Ca^2+^, the Ca^2+^ stocks were emptied in a slow manner. [Na^+^]_i_ was elevated by incubating cells with ouabain in a Na^+^-rich medium prior to the measurements. By blocking the Na^+^-K^+^-ATPase, ouabain causes a gradual rise in intracellular Na^+^ until similar concentrations across the plasma membrane are reached. The activity of both variants of NCX3 was recorded in HEK293T cells expressing an NCX3 variant together with an empty vector (Mock) or the calpain inhibitor calpastatin (CAST). Importantly, our group previously determined the plasma membrane localization of NCX3 in transfected HEK293T cells by cell surface biotinylation. Importantly, NCX3-AC and NCX3-B were both expressed at the plasma membrane in similar amount [30].

**Fig. 2:**
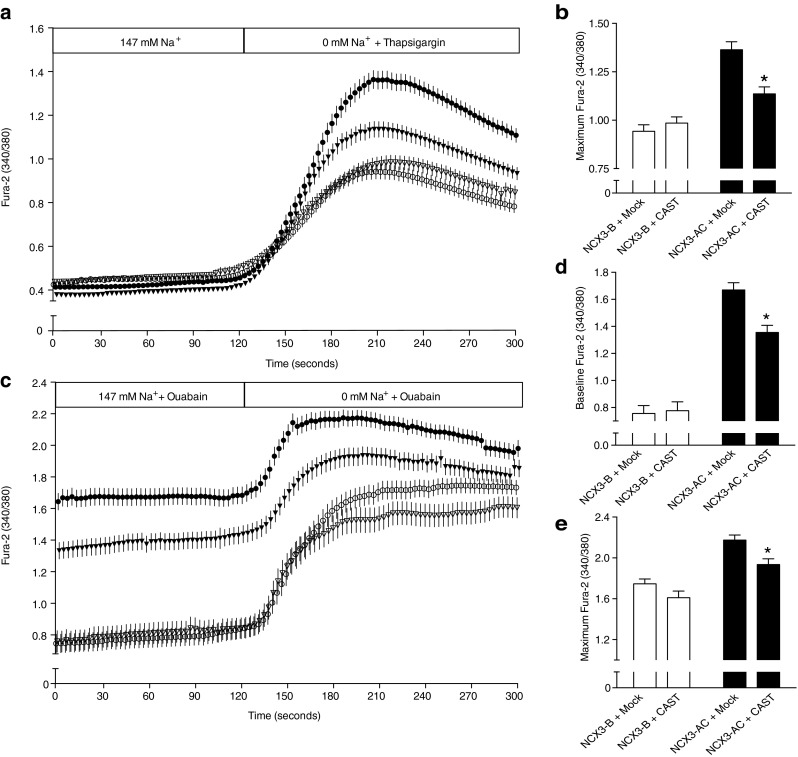
Influence of the calpain inhibitor calpastatin on the reverse mode of exchange of NCX3. The reverse mode of exchange of NCX3 was measured by recording the ratio 340/380 nm in HEK293T cells loaded by Fura-2-AM. Ca^2+^ influx was measured in HEK293T cells expressing NCX3-AC (*filled*) and NCX3-B (*open*) after double transfection with an empty vector (*circle*) or calpastatin (*triangle*). Cells were perfused with a Na^+^-rich medium (147 mM Na^+^). **a** At 120 s, reverse NCX mode was initiated by perfusing with a Na^+^-free medium and depleting the internal calcium stores by applying 1 μM thapsigargin. **b** Mean values of the maximum Fura-2 ratio, shown in **a**, after removal of Na^+^. **c** Reverse NCX mode was recorded after rising intracellular Na^+^ by a 60-min incubation with the Na^+^-K^+^ ATPase inhibitor ouabain and at 120 s by perfusing the cells with a Na^+^-free medium. The 340/380-nm emission ratios are shown representing for each point the mean of the data, after three independent experimental sessions for a number (*n*) of cells (*n* > 71). **d** Baseline of Fura-2 ratio after incubation with ouabain shown in **c. e** Maximum values of Fura-2 ratio after removal of extracellular Na^+^, shown in **c**

In NCX3-B expressing cells, exchange activity remained unchanged by the expression of calpastatin, while the exchange capacity of NCX3-AC was decreased (Fig. 2a, c). Thus, in the reverse mode, the calpastatin expression caused a significant decrease in the maximum Ca^2+^ levels measured in the NCX3-AC expressing cells under Na^+^-free conditions (Fig. 2b, e), regardless of whether intracellular Ca^2+^ or Na^+^ was increased. Furthermore, the expression of the calpain inhibitor significantly diminished the initial Ca^2+^ uptake observed in NCX3-AC expressing cells in the presence of extracellular Na^+^ and ouabain (Fig. 2d).

### Silencing of CAPN1 and the NCX3 Ca^2+^ uptake

**Fig. 3:**
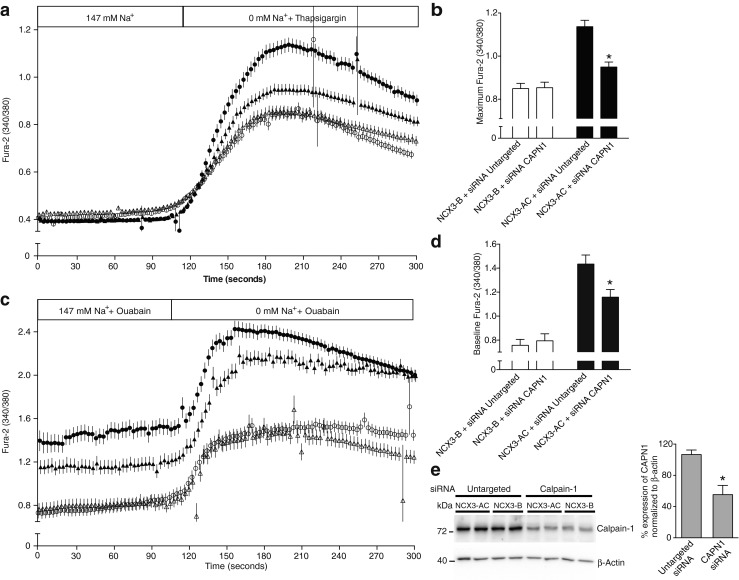
Ca^2+^ influx by NCX3 after silencing of CAPN1. Ca^2+^ influx measured in HEK293T cells loaded with Fura-2-AM and exposed for 72 h to either the CAPN1 siRNA (*triangle*), the untargeted siRNA (*circle*) and simultaneously transfected with NCX3-AC (*filled*), or NCX3-B (*open*). **a** Ca^2+^ uptake was triggered by an increase in intracellular Ca^2+^ caused by exposure to a Na^+^-free medium together with thapsigargin (1 μM) and **c** by an increase in intracellular Na^+^ obtained after 60 min incubation with ouabain (1 μM) followed by the exposure to Na^+^-free medium after 120 s. The ratios are shown representing for each point the mean of the data, after three independent experimental sessions for a number (*n*) of cells (*n* > 70). **b** Mean values of the maximum Fura-2 ratio after addition of thapsigargin (1 μM) shown in **a. d** Mean values of the baseline of Fura-2 ratio after exposure to ouabain, the first 60 s of measurements are averaged. **e** Representative immunoblots of the downregulation of CAPN1 expression 72 h after exposure to siRNA. β-actin is used as a loading control. Percentage of downregulation of CAPN1 was normalized on β-actin expression, and data were measured on four different experiments and for both cells expressing NCX3-B and NCX3-AC

CAPN1, CAPN2, and CAPN3 are the three major isoforms of calpains found in skeletal muscle. CAPN1 and CAPN2 are also ubiquitously expressed and therefore endogenously expressed in HEK293T. Due to their different thresholds of activation by Ca^2+^, CAPN1 was more likely to be the unique isoform activated during our functional assays as its threshold is in the micromolar range, and CAPN2 is activated by Ca^2+^ fluxes in the millimolar range. Incubating the cells with thapsigargin resulted in a mild rise of [Ca^2+^]_i_ in the micromolar range. In a similar manner, the incubation of cells expressing NCX3 with ouabain gradually raised Na^+^ levels and triggered a Ca^2+^ influx that was able to activate CAPN1 [12]. In order to investigate the potential role of CAPN1 on the activity of NCX3 variants, CAPN1 was silenced using siRNA specifically targeting CAPN1 in HEK293T. The expression of CAPN1 was measured by immunoblotting 72 h after transfection (Fig. 3e). CAPN1 has an electrophoretic mobility of 80 kDa [52]. The quantification revealed a significant downregulation of 50 %. As with the cells transfected with calpastatin, the direct silencing of CAPN1 had no effect on NCX3-B activity in reverse mode activated by either [Na^+^]_i_ or [Ca^2+^]_i_ (Fig. 3a, d). Nonetheless, in the reverse mode conditions triggered by intracellular Ca^2+^, silencing CAPN1 significantly decreased the maximum Ca^2+^ uptake of the NCX3-AC expressing cells compared to the untargeted siRNA conditions (Fig. 3b). This effect is also observed when [Na^+^]_i_ is increased, where intracellular Ca^2+^ uptake is significantly decreased during perfusion with a Na^+^-rich medium (Fig. 3d).

### Influence of the proteolytic activity of CAPN3 on NCX3 capacity

**Fig. 4:**
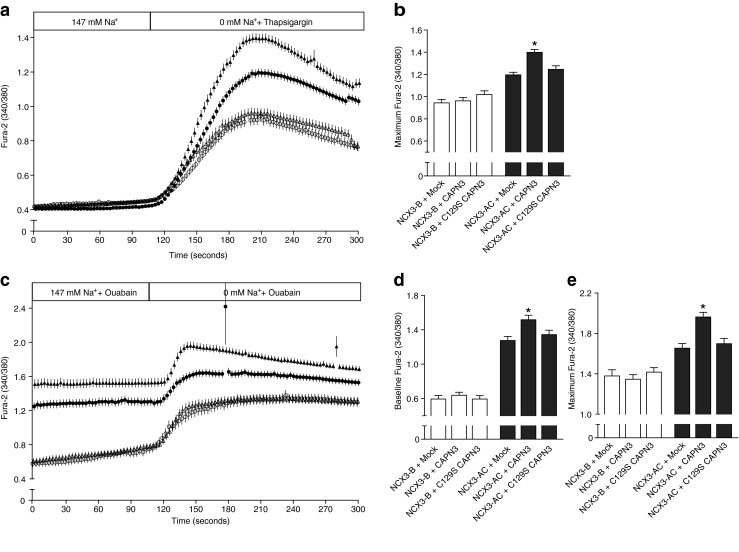
The reverse mode of NCX3 and the skeletal muscle-specific CAPN3. The reverse mode of NCX3 was measured in HEK293T cells by recording the 340/380-nm ratio after loading the cells with Fura-2. Cells were transfected with NCX3-AC (*filled*) and NCX3-B (*open*) after double transfection with an empty vector (*circle*), CAPN3 (*triangle*), or C129S CAPN3, an inactive form of CAPN3. For clarity, the recordings of C129S CAPN3 have not been included in **a** and **c**. The reverse mode was triggered by the removal of extracellular Na^+^ **a**, simultaneously with a rise in intracellular Ca^2+^ by thapsigargin or **c**, after 60 min incubation with ouabain. The ratios are shown representing for each point the mean of the data, after three independent experimental sessions for a number (*n*) of cells (*n* > 47). **b**, **e** Mean values of the maximum Fura-2 ratio after removal of Na^+^ and **d** at the baseline that includes the first 60 s of measurements shown in **a** and **c**, respectively, and the C129S CAPN3

The third isoform of calpain, CAPN3, was not expressed in our cell model and was therefore transfected together with NCX3 variants. The overexpression resulted in an increase in the maximum intracellular Ca^2+^ levels recorded in NCX3-AC expressing cells in both thapsigargin and ouabain conditions (Fig. 4). Additionally, the baseline measured after ouabain incubation was significantly elevated compared to the normal conditions revealing a higher Ca^2+^ uptake in presence of extracellular Na^+^ (Fig. 4d). In both conditions, the exchange capacity of the NCX3-B expressing cells remained unchanged.

Site-directed mutagenesis was used to introduce a C129S mutation in the CAPN3 sequence. This mutation inactivated the catalytic site of CAPN3 [14]. In cells expressing C129S CAPN3, the function of NCX3-B measured in both ouabain and thapsigargin conditions was unchanged, while the increase in Ca^2+^ uptake observed for NCX3-AC was abrogated (Fig. 4b, d, and e).

### Targeting the molecular determinants of the regulation by calpain

**Fig. 5:**
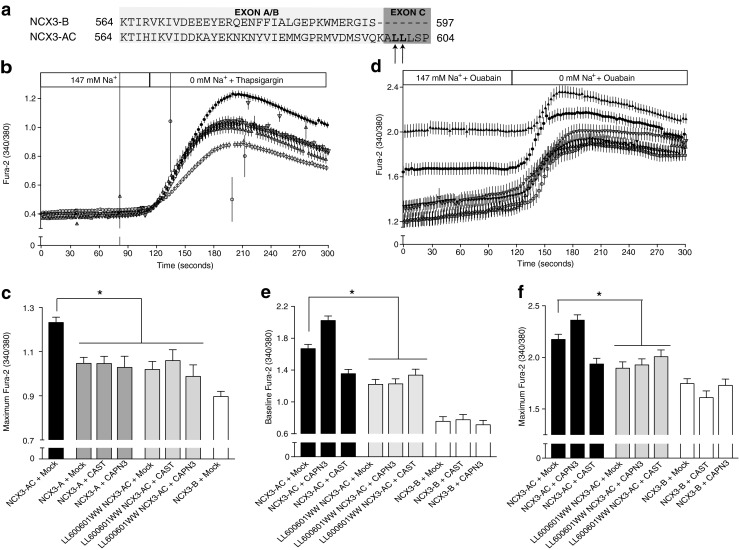
Targeting the calpain cleavage site of NCX3-AC. **a** Alignment of amino acid sequence of the alternatively spliced exons **a**, **b**, and **c** of the two variants NCX3-AC and NCX3-B using ClustalW software. Mutated residues are indicated by *arrows* and missing residues by *dashes*. The predicted calpain cleavage site is located after the second arrow. **b**–**f** 340/380 nm ratios recorded in HEK293T cells transfected loaded with Fura-2-AM during the reverse mode of NCX3 after removal of extracellular Na^+^, **b** and **d** together with the exposure to thapsigargin (1 μM) and **d** to **f**, after 60 min exposure to ouabain (1 μM). **b** Fura-2 ratio of cells expressing NCX3-AC (*black*), NCX3-A (*gray*), and NCX3-B (*white*) together with an empty vector (*circle*), calpastatin (*down-pointing triangle*), and CAPN3 (*up-pointing triangle*). For clarity, the recordings of the mutant LL600601WW NCX3-AC are not shown. **c** Mean values of the maximum Fura-2 ratio after removal of Na^+^ in all recorded conditions. **d** The Ca^2+^ influx has been measured by recording the 340/380-nm ratio in cells loaded with Fura-2 ratio and expressing NCX3-AC (*black*) or LL600601WW NCX3-AC (*gray*) together with an empty vector (*circle*), calpastatin (*down-pointing triangle*), and CAPN3 (*up-pointing triangle*). For clarity, the recordings of NCX3-B variant have not been included. **e**, **f** Mean values of the baseline and maximum Fura-2 ratio, respectively. The baseline represents the mean values for the first 60 s of the measurements. All ratios represent for each point the mean of the data, after three independent experimental sessions for a number (*n*) of cells (*n* > 54)

The data recorded in Figs. 2, 3, and 4 clearly demonstrated that NCX3-AC activity is regulated by different calpains. Additionally, one of the cleavage sites for calpain predicted by the software program GPS-CCD 1.0 [27] is located in the exon C of NCX3-AC, between the Leu-601 and Leu-602 (Fig. 5a). Next, exon C was removed by site-directed mutagenesis. The mutant NCX3-A had a significantly lower Ca^2+^ uptake when incubated with thapsigargin than NCX3-AC (Fig. 5b, c). This decrease corresponded to the observed effect of silencing of CAPN1 and remained significantly higher than NCX3-B activity. Furthermore, expression of CAST or CAPN3 had no significant effect on the Ca^2+^ uptake recorded for NCX3-A expressing cells (Fig. 5b, c), which confirms that the exon C is directly implicated in the regulation by calpain possibly by cleavage.

The positions −1 and −2 of a cleavage site are of major importance for the recognition of the site by calpain [49]. Therefore, the residues Leu-600 and Leu-601 were mutated into Trp-600 and Trp-601, residues carrying the lowest probability of occurrence in a calpain cleavage site [49], and unlikely to be recognized by the calpain. In the reverse mode activated by an increase in [Ca^2+^]_i_, the capacity of the LL600601WW-NCX3-AC mutant was significantly decreased compared to NCX3-AC and similar to NCX3-A (Fig. 5b, c). In a similar manner, during reverse activation by an increase in [Na^+^]_i_, the highest intracellular Ca^2+^ value of LL600601WW-NCX3-AC in Na^+^-free conditions is decreased compared to NCX3-AC expressing cells (Fig. 5d, f). In Na^+^-rich conditions, the exchange capacity of LL600601WW-NCX3-AC mutant as observed by the baseline of Fura-2 during ouabain exposure was decreased compared to NCX3-AC (Fig. 5e), but it was identical to NCX3-AC in the presence of calpastatin while remaining significantly higher than NCX3-B. The LL600601WW-NCX3-AC activity remained unchanged in CAST and CAPN3 conditions in both thapsigargin and ouabain conditions, similarly to NCX3-A. Thereby, the mutation of the two residues abrogated the regulation of the exchanger by both CAPN1 and CAPN3.

## Discussion

The present study unraveled for the first time the influence of the muscle-specific CAPN3 on the activity of NCX3. Furthermore, this study revealed the specificity of CAPN1 and CAPN3 for the variant NCX3-AC, predominant in skeletal muscle, as an increase of the reverse mode of exchange is observed solely for this variant and implicates the presence of the exon C to be responsible for the CAPN effect. First, the muscle-specific CAPN3 elevated the Ca^2+^ uptake performed by NCX3-AC in its reverse mode triggered by a rise in either [Ca^2+^]_i_ or [Na^+^]_i_. Such an increase was absent in NCX3-B expressing cells. The involvement of CAPN3 catalytic activity was further demonstrated by the lack of effect with the inactive form of CAPN3, the C129S mutant. Secondly, the inhibition by calpastatin of multiple proteases such as CAPN1 and CAPN2 decreased Ca^2+^ uptake by NCX3-AC, an effect unique to this variant. The silencing of CAPN1 confirmed this effect, thereby suggesting that CAPN2 and additional intracellular proteases inhibited by calpastatin have no influence on NCX3 activity [2]. Together, these results help to unravel the roles of CAPN1 and CAPN3 in increasing the reverse mode capacity of NCX3.

In the present experiments, the NCX3 activity was triggered by a Na^+^ removal in the extracellular medium, which lead to a strong intracellular Ca^2+^ entry. Such entry provokes CAPN1 autolysis as this process can be rapidly achieved following a strong Ca^2+^ rise, although in diverse cell types, CAPN1 can also be found in its autolyzed form at resting state [6, 35]. In this regard, previous study from our group has shown that Ca^2+^ levels in HEK293T cells expressing NCX3-AC and NCX3-B were unchanged in both cytoplasm and ER at resting state [30]. Therefore, CAPN1 activation in this study is expected to be similar in all conditions.

Additionally, this study identified the molecular determinants of the calpain sensitivity. Among the predicted sites for calpain cleavage, a variant-specific site was predicted in exon C, and the activity of NCX3-A, a mutant missing the exon C, was measured. NCX3-A had an activity similar to the one recorded under CAPN1 inhibition by either calpastatin or siRNA, thereby demonstrating the loss of the calpain sensitivity. The two key residues of the hypothesized calpain cleavage site, Leu-600 and Leu-601, were mutated into Trp-600 and Trp-601, respectively. These mutations diminished the Ca^2+^ uptake performed in the reverse mode of exchange by NCX3-AC. The similarity to NCX3-A activity confirmed the implication of the two residues Leu-600 and Leu-601 in the regulation of NCX3-AC by calpain, which possibly occurs by cleavage. However, the decreased activity of NCX3-AC in the absence of calpain cannot fully explain the differences observed between the variant NCX3-AC and NCX3-B, as the mutations of Leu-600 and Leu-601 are not sufficient to obtain the properties of NCX3-B. The difference between the two variants has been previously attributed to the mutually exclusive exons A and B and their influence on the Ca^2+^-binding domain 2 (CBD2) of the intracellular loop of NCX3. However, the exon C, responsible for the sensitivity to calpain, most likely contributes as well to the functional differences between the two variants. This additional effect of exon C could explain the inability of any of the mutations targeting the CBD2 in exon B of NCX3-B to fully reproduce NCX3-AC activity in the reverse mode activated by intracellular Na^+^ [30].

Calpain recognizes its cleavage site by the primary sequence as well as the tertiary structure, which complicates the identification of a consensus sequence for the calpain cleavage site. Tompa et al. attempted to pinpoint the residues necessary for cleavage in 11 amino acids [49]. The exon C, where a calpain cleavage site is predicted at Leu-602, carries only six residues—ALLLSP—that have a high occurrence in calpain cleavage sites [49]. Here, the investigation of the potential cleavage of NCX3-AC at Leu-602 and detection of its generated fragments would be needed to confirm whether or not the exon C is carrying a cleavage site for calpain. Furthermore, the neighboring sequence at the end of exon A and at the beginning of exon 5 is of importance as well for the recognition by calpain. The point mutation of each residue of this region would provide further information on which residues are necessary for the recognition by calpain.

Interestingly, the three Leucine residues located next to the predicted cleavage site are conserved within the NCX family. NCX2, expressed in the brain, also expresses the exon C of sequence –ALLLNQ-. In NCX1, the exon C of sequence –ALLLNEL- [38] is alternatively spliced. The splice variants of NCX1 carrying the exon C are detected in excitable tissues [24, 28, 38]. Additionally, the cleavage of NCX by the calpain family has only been reported in excitable tissues [3, 4, 50]. Therefore, the implication of the exon C in the regulation of both NCX1 and NCX2 and its potential cleavage by calpain at position Leu-602 warrants further exploration.

The cleavage of NCX3 by CAPN1, leading to a hyperactive exchanger, has been reported previously [37]. The description by Bano et al. of the cleavage sites in neurons did not describe a site located on the exon C [4]. Furthermore, NCX3-B is predominant in neuronal tissues, but in our experiments, NCX3-B was unaffected by CAPN1. This could be explained by the different cellular model used and conditions of experiments that would lead to diverse status of the cells. It has been found that several types of post-translational regulation, such as calmodulin binding [18, 46], phosphorylation [34, 53], and phosphoinositide binding [48], can be involved in protecting the cleavage site of the target protein or favor a specific site of cleavage. From these different studies, some common mechanisms arise. First, the intracellular loop of NCX3 is of significant importance for NCX3 regulation. Secondly, in NCX3 as well as in diverse Ca^2+^ channels [21, 42] and transporters [19, 44], the proteolytic activity of the calpain family goes beyond its role in protein degradation and provides an additional Ca^2+^-dependent regulatory mechanism in order to adjust the Ca^2+^ fluxes of the cells.

In this study, we have shown that CAPN1, CAPN2, and the muscle-specific CAPN3 are expressed in skeletal muscle. Unlike CAPN2, CAPN1 and CAPN3 can be activated under physiological conditions in myofibers. CAPN1, freely present in the sarcoplasm, is autolyzed and activated during the prolonged elevation of [Ca^2+^]_i_ to micromolar levels such as that following eccentric exercise. In these conditions, a disruption of the excitation-contraction (EC) coupling can occur, caused by the decrease in Ca^2+^ release from the SR that could lead to post-exercise fatigue and contribute to the muscle weakness observed in several muscular pathologies [25]. In the myocardium, the CAPN1 activation observed during an ischemic event affects the SR Ca^2+^ content and release capacity leading to a cardiac contractile dysfunction [45]. Therefore, the increased activity of NCX3-AC caused by CAPN1 could either constitute a protective attempt to refill the intracellular Ca^2+^ stores in skeletal muscle and to delay the E-C uncoupling or to compete with the Ca^2+^ re-uptake into the SR by an enhanced Ca^2+^ extrusion. The low rate of [Ca^2+^]_i_ increase during these specific uncoupling events greatly favors the activation of the reverse mode. Furthermore, the capacity of NCX to refill the SR Ca^2+^ levels has been observed in several excitable tissues such as cardiomyocytes [16], slow-twitch fibers [11], and smooth muscle cells [8, 26]. Additionally, both an elevated CAPN1 activity [54] and an increased capacity of exchange by NCX [10] were observed in the *mdx* model of Duchenne muscular dystrophy, where higher sarcoplasmic Ca^2+^ levels were found. Finally, the cleavage of NCX3 by calpain in neurons provoked a significant increase in ER Ca^2+^ content and delayed the caspase activation during neuronal excitotoxicity [37].

Regarding CAPN3, in the myofiber, the protease is tightly bound at the myofibril, and at the triad, a privileged region for Ca^2+^ fluxes responsible for the EC coupling. The conditions necessary for CAPN3 activation are not yet fully understood. Its activation seems to occur from 24 to 48 h following eccentric exercise [31], a phase of intense sarcomeric remodeling where the SR Ca^2+^ content slowly rises again until it reaches normal levels 48 h after exercise [7]. The increase in NCX3 activity following CAPN3 cleavage could, therefore, be involved in the persistent increase in the Ca^2+^ content of the SR during the days following eccentric exercise. This hypothesis is consistent with the Ca^2+^ disturbances [9, 23], such as a low Ca^2+^ release from the SR, observed in the CAPN3 knock-out (KO) fibers mimicking the LGMD2A condition. Additionally, while several potential substrates of CAPN3 appear to be affected by the protease through non-proteolytic action [36], the proteolytically inactive CAPN3 (C129S CAPN3) was not shown to have any effect on NCX3-AC activity, further demonstrating that CAPN3 modulates NCX3-AC through cleavage. It has to be noted that in our study, the CAPN3 is expressed in high levels and freely present in the cytoplasm of the HEK cells, therefore more prone to activation at lower [Ca^2+^]_i_. This might well explain the activation of CAPN3 in our experiments despite the specific requirements for its activation, described by others, in terms of [Ca^2+^]_i_ and exercise. Our study has been performed in HEK cells, as a well-used cell model for the investigation of the capacity of exchange and the ionic regulations of the NCX family [17, 20]. The study of the regulation of NCX3 by calpain in the dense cellular organization of the myofiber would be essential in order to conclude on the role of NCX3 in the Ca^2+^ disturbances of LGMD2A.

In conclusion, a novel target sensitive to the muscle-specific CAPN3 was identified. This sensitivity was found to be restricted to the skeletal muscle variant NCX3-AC. The site located in the exon C was identified as implicated in the regulation of the exchanger by CAPN1 and CAPN3, possibly by a direct cleavage and might be involved with other Ca^2+^ transporters in the Ca^2+^ refilling of the SR, subsequent to the EC uncoupling. The loss of regulation of NCX3 by CAPN3 could therefore be implicated in diverse muscular pathologies such as the LGMD2A.

## References

[1] Al-Qusairi L, Laporte J (2011). T-tubule biogenesis and triad formation in skeletal muscle and implication in human diseases. Skelet Muscle.

[2] Ali MA, Stepanko A, Fan X, Holt A, Schulz R (2012). Calpain inhibitors exhibit matrix metalloproteinase-2 inhibitory activity. Biochem Biophys Res Commun.

[3] Atherton J, Kurbatskaya K, Bondulich M, Croft CL, Garwood CJ, Chhabra R, Wray S, Jeromin A, Hanger DP, Noble W (2014). Calpain cleavage and inactivation of the sodium calcium exchanger-3 occur downstream of Abeta in Alzheimer's disease. Aging Cell.

[4] Bano D, Young KW, Guerin CJ, Lefeuvre R, Rothwell NJ, Naldini L, Rizzuto R, Carafoli E, Nicotera P (2005). Cleavage of the plasma membrane Na+/Ca2+ exchanger in excitotoxicity. Cell.

[5] Branca D, Gugliucci A, Bano D, Brini M, Carafoli E (1999). Expression, partial purification and functional properties of the muscle-specific calpain isoform p94. Eur J Biochem.

[6] Campbell JS, Hallett MB (2015). Active calpain in phagocytically competent human neutrophils: electroinjection of fluorogenic calpain substrate. Biochem Biophys Res Commun.

[7] Chen W, Ruell PA, Ghoddusi M, Kee A, Hardeman EC, Hoffman KM, Thompson MW (2007). Ultrastructural changes and sarcoplasmic reticulum Ca2+ regulation in red vastus muscle following eccentric exercise in the rat. Exp Physiol.

[8] Davis KA, Samson SE, Hammel KE, Kiss L, Fulop F, Grover AK (2009). Functional linkage of Na+-Ca2+-exchanger to sarco/endoplasmic reticulum Ca2+ pump in coronary artery: comparison of smooth muscle and endothelial cells. J Cell Mol Med.

[9] Dayanithi G, Richard I, Viero C, Mazuc E, Mallie S, Valmier J, Bourg N, Herasse M, Marty I, Lefranc G, Mangeat P, Baghdiguian S (2009). Alteration of sarcoplasmic reticulum ca release in skeletal muscle from calpain 3-deficient mice. Int J Cell Biol.

[10] Deval E, Levitsky DO, Marchand E, Cantereau A, Raymond G, Cognard C (2002). Na(+)/Ca(2+) exchange in human myotubes: intracellular calcium rises in response to external sodium depletion are enhanced in DMD. Neuromuscul Disord.

[11] Germinario E, Esposito A, Midrio M, Peron S, Palade PT, Betto R, Danieli-Betto D (2008). High-frequency fatigue of skeletal muscle: role of extracellular Ca(2+). Eur J Appl Physiol.

[12] Harwood SM, Allen DA, Chesser AM, New DI, Raftery MJ, Yaqoob MM (2003). Calpain is activated in experimental uremia: is calpain a mediator of uremia-induced myocardial injury?. Kidney Int.

[13] Hata S, Abe M, Suzuki H, Kitamura F, Toyama-Sorimachi N, Abe K, Sakimura K, Sorimachi H (2010). Calpain 8/nCL-2 and calpain 9/nCL-4 constitute an active protease complex, G-calpain, involved in gastric mucosal defense. PLoS Genet.

[14] Herasse M, Ono Y, Fougerousse F, Kimura E, Stockholm D, Beley C, Montarras D, Pinset C, Sorimachi H, Suzuki K, Beckmann JS, Richard I (1999). Expression and functional characteristics of calpain 3 isoforms generated through tissue-specific transcriptional and posttranscriptional events. Mol Cell Biol.

[15] Hoenderop JG, Hartog A, Stuiver M, Doucet A, Willems PH, Bindels RJ (2000). Localization of the epithelial Ca(2+) channel in rabbit kidney and intestine. J Am Soc Nephrol.

[16] Hove-Madsen L, Tort L (2001). Characterization of the relationship between Na+-Ca2+ exchange rate and cytosolic calcium in trout cardiac myocytes. Pflugers Arch.

[17] Hurtado C, Prociuk M, Maddaford TG, Dibrov E, Mesaeli N, Hryshko LV, Pierce GN (2006). Cells expressing unique Na+/Ca2+ exchange (NCX1) splice variants exhibit different susceptibilities to Ca2+ overload. Am J Physiol Heart Circ Physiol.

[18] Iwamoto N, Lu R, Tanaka N, Abe-Dohmae S, Yokoyama S (2010). Calmodulin interacts with ATP binding cassette transporter A1 to protect from calpain-mediated degradation and upregulates high-density lipoprotein generation. Arterioscler Thromb Vasc Biol.

[19] James P, Vorherr T, Krebs J, Morelli A, Castello G, McCormick DJ, Penniston JT, De Flora A, Carafoli E (1989). Modulation of erythrocyte Ca2+-ATPase by selective calpain cleavage of the calmodulin-binding domain. J Biol Chem.

[20] John SA, Ribalet B, Weiss JN, Philipson KD, Ottolia M (2011). Ca2+-dependent structural rearrangements within Na+-Ca2+ exchanger dimers. Proc Natl Acad Sci U S A.

[21] Kaczmarek JS, Riccio A, Clapham DE (2012). Calpain cleaves and activates the TRPC5 channel to participate in semaphorin 3A-induced neuronal growth cone collapse. Proc Natl Acad Sci U S A.

[22] Keira Y, Noguchi S, Minami N, Hayashi YK, Nishino I (2003). Localization of calpain 3 in human skeletal muscle and its alteration in limb-girdle muscular dystrophy 2A muscle. J Biochem.

[23] Kramerova I, Kudryashova E, Wu B, Ottenheijm C, Granzier H, Spencer MJ (2008). Novel role of calpain-3 in the triad-associated protein complex regulating calcium release in skeletal muscle. Hum Mol Genet.

[24] Kuroda H, Sobhan U, Sato M, Tsumura M, Ichinohe T, Tazaki M, Shibukawa Y (2013). Sodium-calcium exchangers in rat trigeminal ganglion neurons. Mol Pain.

[25] Lamb GD (2009). Mechanisms of excitation-contraction uncoupling relevant to activity-induced muscle fatigue. Appl Physiol Nutr Metab.

[26] Lemos VS, Poburko D, Liao CH, Cole WC, van Breemen C (2007). Na+ entry via TRPC6 causes Ca2+ entry via NCX reversal in ATP stimulated smooth muscle cells. Biochem Biophys Res Commun.

[27] Liu Z, Cao J, Gao X, Ma Q, Ren J, Xue Y (2011). GPS-CCD: a novel computational program for the prediction of calpain cleavage sites. PLoS One.

[28] Long Y, Wang WP, Yuan H, Ma SP, Feng N, Wang L, Wang XL (2013). Functional comparison of the reverse mode of Na+/Ca2+ exchangers NCX1.1 and NCX1.5 expressed in CHO cells. Acta Pharmacol Sin.

[29] McDermott JR, Mantle D, Biggins JA, Kidd AM, Davison K, Lauffart B, Pennington RJ (1985). Specificity of neuropeptide degradation by two calcium-activated neutral proteases from human skeletal muscle. Life Sci.

[30] Michel LY, Verkaart S, Koopman WJ, Willems PH, Hoenderop JG, Bindels RJ (2014). Function and regulation of the Na+-Ca2+ exchanger NCX3 splice variants in brain and skeletal muscle. J Biol Chem.

[31] Murphy RM, Goodman CA, McKenna MJ, Bennie J, Leikis M, Lamb GD (2007). Calpain-3 is autolyzed and hence activated in human skeletal muscle 24 h following a single bout of eccentric exercise. J Appl Physiol (1985).

[32] Murphy RM, Lamb GD (2009). Calpain-3 is activated following eccentric exercise. J Appl Physiol (1985).

[33] Murphy RM, Verburg E, Lamb GD (2006). Ca2+ activation of diffusible and bound pools of mu-calpain in rat skeletal muscle. J Physiol.

[34] Nicolas G, Fournier CM, Galand C, Malbert-Colas L, Bournier O, Kroviarski Y, Bourgeois M, Camonis JH, Dhermy D, Grandchamp B, Lecomte MC (2002). Tyrosine phosphorylation regulates alpha II spectrin cleavage by calpain. Mol Cell Biol.

[35] Noma H, Kato T, Fujita H, Kitagawa M, Yamano T, Kitagawa S (2009). Calpain inhibition induces activation of the distinct signalling pathways and cell migration in human monocytes. Immunology.

[36] Ojima K, Ono Y, Ottenheijm C, Hata S, Suzuki H, Granzier H, Sorimachi H (2011). Non-proteolytic functions of calpain-3 in sarcoplasmic reticulum in skeletal muscles. J Mol Biol.

[37] Pannaccione A, Secondo A, Molinaro P, D'Avanzo C, Cantile M, Esposito A, Boscia F, Scorziello A, Sirabella R, Sokolow S, Herchuelz A, Di Renzo G, Annunziato L (2012). A new concept: Abeta1-42 generates a hyperfunctional proteolytic NCX3 fragment that delays caspase-12 activation and neuronal death. J Neurosci : Off J Soc Neurosci.

[38] Quednau BD, Nicoll DA, Philipson KD (1997). Tissue specificity and alternative splicing of the Na+/Ca2+ exchanger isoforms NCX1, NCX2, and NCX3 in rat. Am J Physiol.

[39] Richard I, Broux O, Allamand V, Fougerousse F, Chiannilkulchai N, Bourg N, Brenguier L, Devaud C, Pasturaud P, Roudaut C (1995). Mutations in the proteolytic enzyme calpain 3 cause limb-girdle muscular dystrophy type 2A. Cell.

[40] Richard I, Roudaut C, Saenz A, Pogue R, Grimbergen JE, Anderson LV, Beley C, Cobo AM, de Diego C, Eymard B, Gallano P, Ginjaar HB, Lasa A, Pollitt C, Topaloglu H, Urtizberea JA, de Visser M, van der Kooi A, Bushby K, Bakker E, Lopez de Munain A, Fardeau M, Beckmann JS (1999). Calpainopathy—a survey of mutations and polymorphisms. Am J Hum Genet.

[41] Sacchetto R, Margreth A, Pelosi M, Carafoli E (1996). Colocalization of the dihydropyridine receptor, the plasma-membrane calcium ATPase isoform 1 and the sodium/calcium exchanger to the junctional-membrane domain of transverse tubules of rabbit skeletal muscle. Eur J Biochem.

[42] Sandoval A, Oviedo N, Tadmouri A, Avila T, De Waard M, Felix R (2006). Two PEST-like motifs regulate Ca2+/calpain-mediated cleavage of the CaVbeta3 subunit and provide important determinants for neuronal Ca2+ channel activity. Eur J Neurosci.

[43] Secondo A, Staiano RI, Scorziello A, Sirabella R, Boscia F, Adornetto A, Valsecchi V, Molinaro P, Canzoniero LM, Di Renzo G, Annunziato L (2007). BHK cells transfected with NCX3 are more resistant to hypoxia followed by reoxygenation than those transfected with NCX1 and NCX2: possible relationship with mitochondrial membrane potential. Cell Calcium.

[44] Shoshan-Barmatz V, Weil S, Meyer H, Varsanyi M, Heilmeyer LM (1994). Endogenous, Ca(2+)-dependent cysteine-protease cleaves specifically the ryanodine receptor/Ca2+ release channel in skeletal muscle. J Membr Biol.

[45] Singh RB, Chohan PK, Dhalla NS, Netticadan T (2004). The sarcoplasmic reticulum proteins are targets for calpain action in the ischemic-reperfused heart. J Mol Cell Cardiol.

[46] Sivanandam A, Murthy S, Chinnakannu K, Bai VU, Kim SH, Barrack ER, Menon M, Reddy GP (2011). Calmodulin protects androgen receptor from calpain-mediated breakdown in prostate cancer cells. J Cell Physiol.

[47] Sorimachi H, Hata S, Ono Y (2011). Impact of genetic insights into calpain biology. J Biochem.

[48] Sprague CR, Fraley TS, Jang HS, Lal S, Greenwood JA (2008). Phosphoinositide binding to the substrate regulates susceptibility to proteolysis by calpain. J Biol Chem.

[49] Tompa P, Buzder-Lantos P, Tantos A, Farkas A, Szilagyi A, Banoczi Z, Hudecz F, Friedrich P (2004). On the sequential determinants of calpain cleavage. J Biol Chem.

[50] Wanichawan P, Hafver TL, Hodne K, Aronsen JM, Lunde IG, Dalhus B, Lunde M, Kvaloy H, Louch WE, Tonnessen T, Sjaastad I, Sejersted OM, Carlson CR (2014). Molecular basis of calpain cleavage and inactivation of the sodium-calcium exchanger 1 in heart failure. J Biol Chem.

[51] Whelan JA, Russell NB, Whelan MA (2003). A method for the absolute quantification of cDNA using real-time PCR. J Immunol Methods.

[52] Yoshimura N, Kikuchi T, Sasaki T, Kitahara A, Hatanaka M, Murachi T (1983). Two distinct Ca2+ proteases (calpain I and calpain II) purified concurrently by the same method from rat kidney. J Biol Chem.

[53] Zhang S, Kim TS, Dong Y, Kanazawa S, Kawaguchi M, Gao N, Minato H, Takegami T, Nojima T, Asai K, Miura Y (2012). AT motif binding factor 1 (ATBF1) is highly phosphorylated in embryonic brain and protected from cleavage by calpain-1. Biochem Biophys Res Commun.

[54] Zhao X, Moloughney JG, Zhang S, Komazaki S, Weisleder N (2012). Orai1 mediates exacerbated Ca(2+) entry in dystrophic skeletal muscle. PLoS One.

